# The Structural Pathway of Interleukin 1 (IL-1) Initiated Signaling Reveals Mechanisms of Oncogenic Mutations and SNPs in Inflammation and Cancer

**DOI:** 10.1371/journal.pcbi.1003470

**Published:** 2014-02-13

**Authors:** Saliha Ece Acuner Ozbabacan, Attila Gursoy, Ruth Nussinov, Ozlem Keskin

**Affiliations:** 1 Center for Computational Biology and Bioinformatics and College of Engineering, Koc University, Sariyer Istanbul, Turkey; 2 Cancer and Inflammation Program, Leidos Biomedical Research, Inc., National Cancer Institute, Frederick National Laboratory, Frederick, Maryland, United States of America; 3 Sackler Inst. of Molecular Medicine, Department of Human Genetics and Molecular Medicine, Sackler School of Medicine, Tel Aviv University, Tel Aviv, Israel; McGill University, Canada

## Abstract

Interleukin-1 (IL-1) is a large cytokine family closely related to innate immunity and inflammation. IL-1 proteins are key players in signaling pathways such as apoptosis, TLR, MAPK, NLR and NF-κB. The IL-1 pathway is also associated with cancer, and chronic inflammation increases the risk of tumor development via oncogenic mutations. Here we illustrate that the structures of interfaces between proteins in this pathway bearing the mutations may reveal how. Proteins are frequently regulated via their interactions, which can turn them ON or OFF. We show that oncogenic mutations are significantly at or adjoining interface regions, and can abolish (or enhance) the protein-protein interaction, making the protein constitutively active (or inactive, if it is a repressor). We combine known structures of protein-protein complexes and those that we have predicted for the IL-1 pathway, and integrate them with literature information. In the reconstructed pathway there are 104 interactions between proteins whose three dimensional structures are experimentally identified; only 15 have experimentally-determined structures of the interacting complexes. By predicting the protein-protein complexes throughout the pathway via the PRISM algorithm, the structural coverage increases from 15% to 71%. *In silico* mutagenesis and comparison of the predicted binding energies reveal the mechanisms of how oncogenic and single nucleotide polymorphism (SNP) mutations can abrogate the interactions or increase the binding affinity of the mutant to the native partner. Computational mapping of mutations on the interface of the predicted complexes may constitute a powerful strategy to explain the mechanisms of activation/inhibition. It can also help explain how an oncogenic mutation or SNP works.

## Author Summary

Structural pathways are important because they provide insight into signaling mechanisms; help understand the mechanism of disease-related mutations; and help in drug discovery. While extremely useful, common pathway diagrams lacking structural data are unable to provide mechanistic insight to explain oncogenic mutations or SNPs. Here we focus on the construction of the IL-1 structural pathway and map oncogenic mutations and SNPs to complexes in this pathway. Our results indicate that computational modeling of protein-protein interactions on a large scale can provide accurate, structural atom-level detail of signaling pathways in the human cell and help delineate the mechanism through which a mutation leads to disease. We show that the mutations either thwart the interactions, activating the proteins even in their absence or stabilize them, leading to the same uncontrolled outcome. Computational mapping of mutations on the interface of the predicted complexes may constitute an effective strategy to explain the mechanisms of mutations- constitutive activation or deactivation.

## Introduction

Interleukin-1 (IL-1) is a large family of cytokines (small cell signaling proteins) that mediate innate immune responses to defend the host against pathogens. The IL-1 family has 11 member proteins (IL-1F1 to IL-1F11) and they are encoded by 11 distinct human and mouse genes [1]–[3]. The first discovered family members, IL-1α (newly named IL-1F1) and IL-1β (IL-1F2), are secreted by macrophages and epithelial cells in response to pathogens and have strong proinflammatory properties leading to fever (affecting hypothalamus) and activation of T cells and macrophages.

IL-1 family members have been intensely studied (especially IL-1α and IL-1β) unraveling their roles in a number of autoinflammatory diseases [4]–[6]. Signaling initiated by the IL-1 cytokines increases the expression of adhesion factors on endothelial cells resulting in immune cells (such as phagocytes and lymphocytes) migration to the site of infection. The autoinflammatory disease is a class of chronic inflammation with increased secretion of active IL-1β, thus blocking IL-1β is therapeutically beneficial [7].

**Figure 1:**
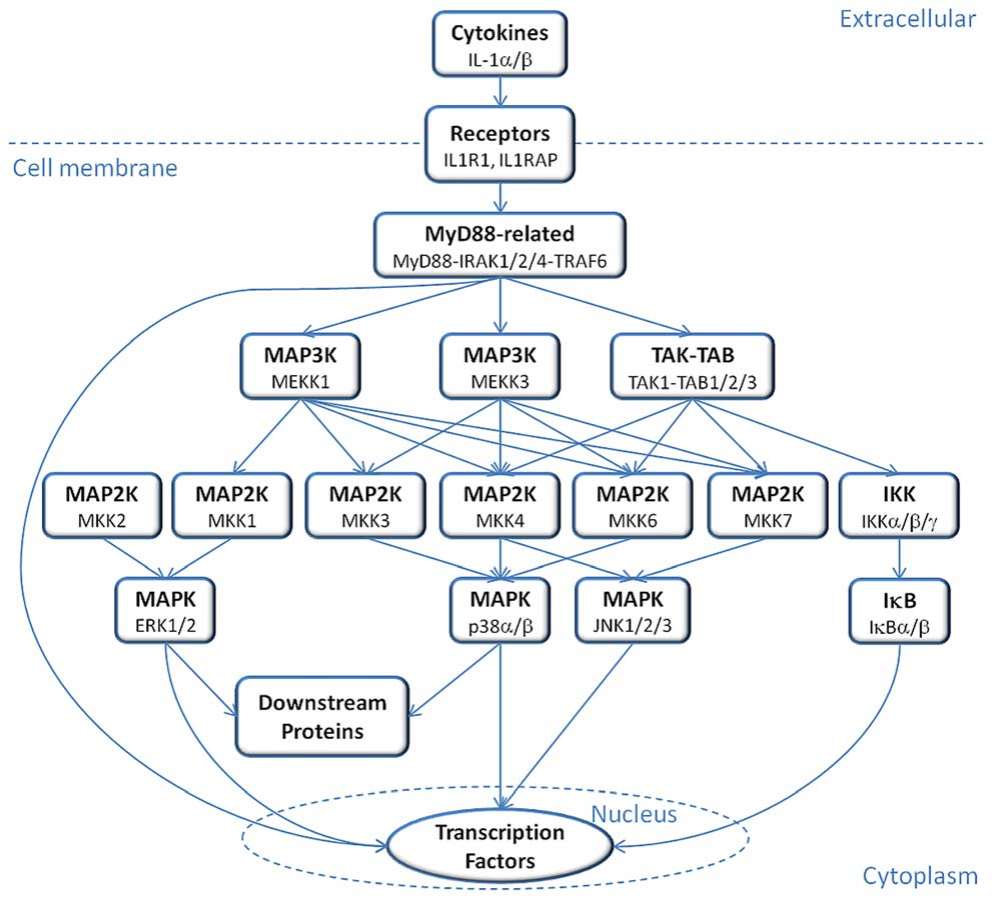
IL-1 signaling pathway diagram. In this simplified diagram of the IL-1 signaling pathway, the signal initiates by the recognition of cytokines by IL-1 receptors and propagates via multiple sub-pathways involving family homologs or alternate pathways to activate transcription factors downstream.

**Figure 2:**
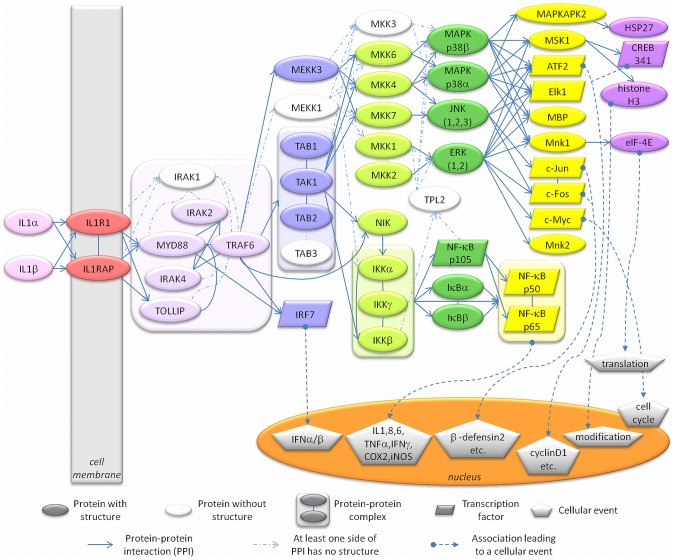
IL-1 signaling pathway reconstructed by combining related pathways and information from the literature. This detailed map of IL-1 signaling presents the protein-protein interactions and the resulting cellular events. The colored nodes represent proteins having experimentally identified 3D structures and the white nodes are the proteins without 3D structures. The edges represent protein-protein interactions (straight/dashed arrows relate to available/unavailable 3D structures of proteins) or associations leading to cellular events such as cell cycle or gene expression (dashed arrows beginning with circular heads).

IL-1α and IL-1β can induce mRNA expression of hundreds of genes, including themselves (a positive-feedback loop), and their gene regulatory actions are conducted via a conserved signaling pathway [8]. Signal propagation mainly depends on mitogen-activated protein kinases (MAPKs), MAPK kinases (MKK/MAP2Ks), MKK kinases (MKKK/MAP3K/MEKKs) and the downstream proteins of MAPKs, finally leading to activation of transcription factors that regulate the expression of host defense proteins (Figure 1). The signal initiates by binding of IL-1α or IL-1β ligands to type I receptor (IL-1R1) and propagates with the help of the co-receptor IL-1 receptor accessory protein (IL-1RAP), forming a trimeric complex [9]. In this trimeric complex, the Toll- and IL-1R–like (TIR) domains on the cytoplasmic regions of IL-1R1 and IL-1RAP receptors get close to each other resulting in the recruitment of myeloid differentiation primary response gene 88 (MYD88), Toll-interacting protein (TOLLIP) [7] and IL-1 receptor-associated kinase 4 (IRAK4) [10], [11]. A stable complex is formed between IL-1, IL-1R1, IL-1RAP, MYD88 and IRAK4 [10]. MYD88 binding triggers phosphorylation of IL-1 receptor-associated kinases IRAK4, IRAK2 and IRAK1, leading to the recruitment and oligomerization of tumor necrosis factor-associated factor 6 (TRAF6) [12]–[14]. TRAF6 and phosphorylated IRAK1 and IRAK2 dissociate and migrate to the membrane to associate with TGF-β-activated kinase 1 (TAK1) and TAK1-binding proteins TAB1 and TAB2 [7]. The TAK1-TAB1-TAB2-TRAF6 complex migrates back to the cytosol, where TRAF6 is ubiquitinated and TAK1 is phosphorylated [7]. From this point, the signal can propagate via two main paths: IKK – IκB – NF-κB and/or MKK – MAPK/JNK/ERK (Figure 2). In the first path, phosphorylated TAK1 activates the inhibitor of nuclear factor kappa-B kinase subunit beta (IKKβ) and activated IKKβ phosphorylates the nuclear factor kappa-B inhibitor (IκB) which gets degraded so that nuclear factor kappa-B kinase (NF-κB) is released and migrates to the nucleus [7]. TAK1 can also activate mitogen-activated kinases (MAPK) p38, c-Jun N-terminal kinases (JNK) and extracellular signal-regulated kinases (ERK) by interacting with MAP kinase kinase (MKK) proteins. Downstream in this path, are transcription factors such as c-Jun, c-Fos, c-Myc and ATF2.

MAP kinase signaling pathways, which are conserved among eukaryotes, mediate cellular events triggered by extracellular signals such as cytokine binding [15] and they are essential for IL-1 signaling (Figures 1 and 2). This pathway builds upon a triple kinase cascade consisting of a MAP kinase kinase kinase (MKKK/MEKK), a MAP kinase kinase (MKK/MEK) and a MAP kinase (MAPK) and these kinases sequentially phosphorylate and activate each other [15]. The JNK and p38 MAP kinases, called stress activated MAP kinases, have roles in tumor suppression and can be both directly phosphorylated and activated by MKK4, which is also a tumor suppressor [16]–[18]. The successive activation mechanism takes place as follows: MEKK interacts with inactive MKK and phosphorylates it; the complex dissociates, releasing the free and active MKK, which is ready to interact with inactive JNK to activate it [15]. Activation of JNK leads to disruption of the MKK-JNK interaction, freeing the active JNK to phosphorylate its downstream targets. There are several mechanisms through which stress activated MAP kinases regulate tumor suppression, including promoting apoptosis (p53, Bax, Bim/Bmf), inhibiting proteins that inhibit apoptosis (Bcl2, Bcl-XL, 14-3-3, Mcl-1), inhibiting tumor development (TGF-β1) and tumor growth (CDC25, CyclinD1/CDK4) [16]. Somatic mutations were identified in the JNK pathway via large-scale sequencing analyses of human tumor cells [19], [20], and JNK3 encoding gene (*MAPK10*) has been speculated to be a putative tumor suppressor gene as almost half of the brain tumors that were examined included mutations [21]. ERK1 and ERK2, the other members of MAPK family, are also upregulated in tumors [22].

Recently, inflammation has been related to cancer [23]–[25]. Cancers are mostly due to somatic mutations and environmental factors, and chronic inflammation is implicated by most of these risk factors [26]. Chronic inflammation, due to autoimmune diseases or infections, causes tumor development via several mechanisms, including oncogenic mutation induction [26]. Oncogenic mutations and single nucleotide polymorphisms (SNPs) are key players in inflammation-related cancers and it is crucial to map the mutations/SNPs on the corresponding 3D structures of the proteins to gain insight into how they affect protein function [27], [28]. SNPs that cause diseases, if not in the core of the protein, are frequently located in protein-protein interface regions rather than elsewhere on the surface [26], [28]. Structural knowledge can clarify the conformational and functional impact of the mutation/SNP on the protein [27]–[29]. The effect of a functional mutation can be expressed by a change in the specificity of the interactions between a mutated protein and its partners [30]. Quantitatively, a mutation changes the binding free energies of the mutant's interactions with its partners with respect to the free energies of its interactions in the native form [30]. The functional impact of the mutation on the specificity differs. The mutation can destabilize the protein and/or its interaction, leading to ‘loss-of-function’; or can lead to a change in the specificity of protein-partner interactions, resulting in a ‘gain-of-function’, or can gain new binding partners and hence a new biological function, i.e. result in a ‘switch-of-function’ [30].

Two recent studies used structural pathways for mapping mutations on protein-protein interfaces, one on smaller scale pathways [27] and the other on large scale [28]. Mosca *et al.* [27] mapped mutations onto proteins as an application of their useful computational modeling technique with the data limited to the Kyoto Encyclopedia of Genes and Genomes (KEGG) complement cascade pathway and the interactions of complement component 3 (C3) and complement factor H (CFH) which it includes. In a pioneering work, Wang *et al.* [28] explored genotype-phenotype relationships on a large scale. They systematically examined thousands of mutations and mapped them onto interaction interfaces and experimentally validating their predictions for the MLH1-PMS2, WASP-CDC42 and TP63–TP73 interactions. These and other studies emphasized the need for computational methods for large-scale interactome studies [31], [32]. We propose a method similar to ones used in the works of Mosca *et al.* [27] and Wang *et al.* [28], while introducing the advantage of *in silico* mutagenesis to observe the effects of mutations on protein-protein interactions on a large scale.

**Figure 3:**
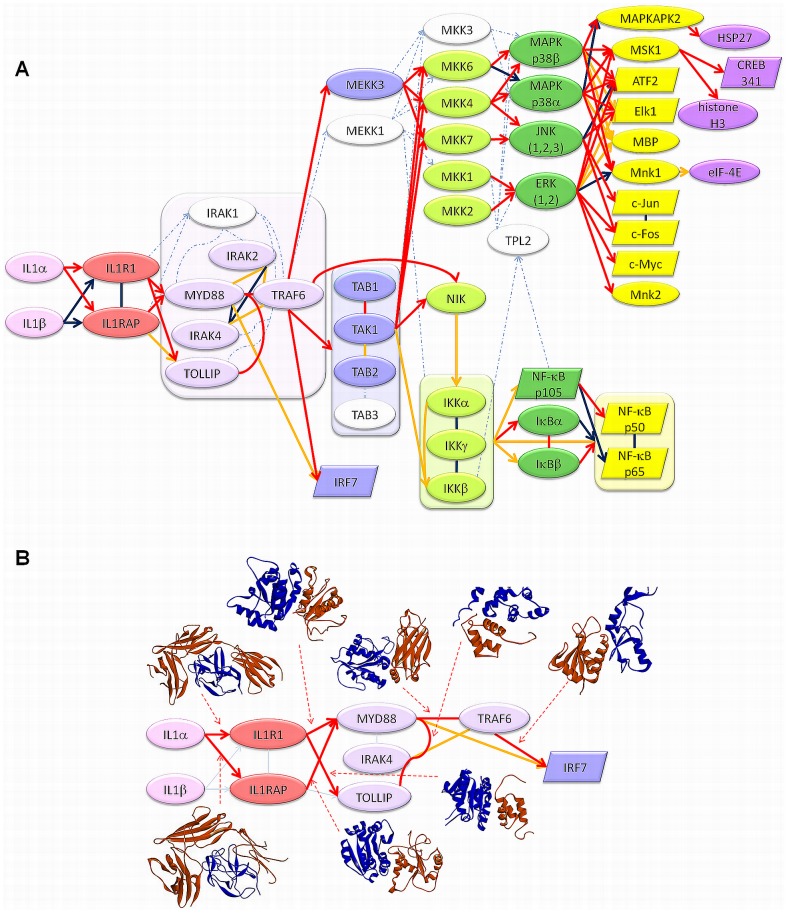
Structures of protein-protein complexes mapped to the IL-1 signaling pathway. **A.** Overall distribution of experimental and predicted complexes on the pathway. Dark blue interactions represent the experimentally determined complex structures in the PDB, red interactions represent the predicted complexes with predicted binding energies lower than −10 energy units and yellow interactions represent the interactions for which neither experimental nor computational data exists. **B.** Predicted structures (Template PDB code+Target PDB codes+Energy value): IL1α-IL1R1 (1itbAB 2l5x 4depB −71.92); IL1α-IL1RAP (1itbAB 2l5x 4depC −49.25); IL1R1-MYD88 (1gylAB IL1R1 (model) 2z5v −23.8); IL1R1-TOLLIP (1oh0AB IL1R1 (model) 1wgl −18.85); IL1RAP-MYD88 (1p65AB IL1RAP (model) 2z5v −31.72); MYD88-TOLLIP (1yrlAC 3mopA 1wgl −11.04); MYD88-TRAF6 (1vjlAB 2js7 1lb6 −37.01); TRAF6-IRF7 (1g8tAB 2o61 3hct −25.83). The blue color represents the proteins that precede its partners in the information flow.

Here, we construct the IL-1 signaling pathway by combining the related pathways and information from the literature [8], [33]–[35] (Figure 2). We observe that there are approximately 100 interactions between proteins that have experimentally identified 3D structures. However, only 15 of the interactions have structures of protein-protein complexes (Figures 3A and S1) in the Protein Data Bank (PDB, http://www.rcsb.org/pdb/) [36]. The structural coverage of the pathway is under 20z%. Our major aim is to expand the structural apoptosis pathway [37], and map oncogenic mutations and SNPs to reveal their mechanism.

## Results/Discussion

### Reconstruction and structural analysis of IL-1 pathway

We are able to increase the structural coverage from 15% up to 71% by predicting the protein-protein complexes throughout the pathway using the PRISM algorithm [38]–[40], such that the number of predicted structures of protein-protein complexes that have binding energies lower than −10 energy units is 74 out of 104 interactions (Figure 3). The predicted structures of complexes are also compared with the 15 available PDB structures of the complexes in the pathway. Except for three where the proteins interact with peptides, all PDB structures could be successfully reproduced via the predictions (Table S1).

### Mapping and analysis of SNPs and oncogenic mutations in the IL-1 pathway

A large scale analysis of the distribution of oncogenic mutations and SNPs in the predicted and experimental structures of protein-protein complexes in the IL-1 pathway indicated that both oncogenic mutations and SNPs correspond significantly to interface regions and their ‘nearby’ residues (Tables 1–4 and Table S2) with p-values 0.00013 and 0.0009, respectively. Nearby residues are defined as the residues that are at most 6 Å away from interface residues in three dimensional space. Along similar lines, David *et al.* showed that SNPs, if not in the protein core, are more likely to occur at the protein-protein interfaces rather than the remaining surface [41].

**Table 1:** The distribution of oncogenic mutations and SNPs on PDB structures of target proteins in IL-1 pathway.

Protein	PDB Code	COSMIC	COSMIC Surface	LS-SNP	LS-SNP Surface	Total Residues	Surface Residues
ATF2, CREB	1t2kD	1	0	1	1	61	61
ATF2, CREB	4h36B	0	0	0	0	8	8
c-Fos, FOS	1fosE	4	4	2	2	60	60
c-Jun, JUN	1jnmA, 1fosF	4	4	1	1	57	57
c-Myc, MYC	1nkpA	8	7	0	0	88	81
ELK1	1duxC	7	5	0	0	88	61
ERK1, MAPK3	2zoqA	14	10	11	9	351	209
ERK2,MAPK2	1wzyA, 3teiA, 4fv4A, 4fv7A, 1tvoA, 4fv0A, 2y9qA	26	17	3	3	365	199
histone H3.3	4hgaB	0	0	6	6	93	85
HSP27	3q9qA	2	1	9	9	79	67
NFKBIA	1nfiE, 1iknD	0	0	8	6	215	142
NFKBIB	1oy3D	0	0	0	0	220	130
IKBKA, CHUK, IKBKB	3brtA, 3brvA	0	0	8	8	39	39
IKBKG	3brvB, 3brtB	0	0	1	1	62	61
IL1A	2l5xA	6	3	11	8	151	102
IL1R1	4depB	18	13	29	21	296	216
IL1RAP	4depC	16	13	14	9	288	201
IRAK2	3mopK	3	1	11	5	93	65
IRAK4	3mopG	2	2	7	5	107	76
IRF7	2o61A	0	0	0	0	498	351
JNK1, MAPK8	3o17A, 1ukhA, 3eljA	30	18	8	7	366	213
JNK2, MAPK9	3e7oA, 3npcA	30	18	9	8	357	211
JNK3	3fv8A, 2zduA, 2r9sA, 4h36	2	1	8	4	356	203
MAPKAPK2	2jboA, 2onlC	22	16	13	11	317	211
MAPK14	1kv1A, 2bajA, 3odyX, 3queA, 1w82A, 2y8oA, 2onlA	0	0	30	24	351	211
MAPK11	3gc9A, 3gp0A, 3gc8A	0	0	26	21	347	213
MEKK3, MAP3K3	2pphA, 2jrhA, 2c60A	6	4	3	3	93	76
MKK1, MAP2K1	2p55A	31	17	6	6	289	181
MKK2, MAP2K2	1s9iA	17	12	12	10	303	182
MKK4, MAP2K4	3aloA, 3alnA, 3vutA	56	40	6	4	289	190
MKK6, MAP2K6	3vn9A, 3fmeA	25	19	7	6	291	174
MKK6, MAP2K6	2y8oB	0	0	0	0	8	8
MKK7, MAP2K7	2dylA	29	22	6	3	272	172
MNK1, MKNK1	2hw6A	0	0	16	10	242	145
MNK1, MKNK1	2y9qB	0	0	0	0	17	17
MNK2, MKNK2	2ac3A	0	0	9	7	277	191
MSK1, RPS6KA5	3kn5A, 3kn6A	23	12	14	10	282	174
MSK1, RPS6KA5	1vzoA	20	14	15	12	324	188
MYD88	2js7A, 2z5vA	22	14	0	0	160	106
MYD88	3mopA	7	7	0	0	105	74
NF-kBp105	3gutB	0	0	9	7	312	198
NFKB1	1svcP, 1nfiB	0	0	8	6	311	198
NFKB3, RELA	1nfiA, 3gutA	0	0	12	11	295	208
NIK, MAP3K14	4g3dA, 4dn5A	14	7	5	4	331	194
TAB1	2j4oA	25	18	21	16	355	207
TAK1	2yiyA, 2evaA	21	15	8	4	295	181
TOLLIP	1wglA	3	3	5	3	59	51
TRAF6	1lb4A, 1lb5A, 1lb6A,	12	11	3	2	155	112
TRAF6	3hcsA, 3hctA, 2eciA	13	11	9	7	157	119
IL1B	4depA	16	12	4	2	151	100
IL1R1 (model)	-	0	0	0	0	157	106
IL1RAP (model)	-	0	0	0	0	145	96
	**TOTAL**	**535**	**371**	**394**	**302**	**10988**	**7181**

**Table 2:** The distribution of oncogenic mutations and SNPs on structures of interacting protein-protein complexes in IL-1 pathway.

Interacting Protein Pairs		COSMIC on Interface	COSMIC on Nearby	LS-SNP on Interface	LS-SNP on Nearby	Interacting Protein Pairs		COSMIC on Interface	COSMIC on Nearby	LS-SNP on Interface	LS-SNP on Nearby
1nfiE	1nfiA	0	0	1	1	1ukh	1fosF	3	3	0	0
2pph	1lb6	2	0	0	1	2o61	3hct	2	2	0	0
4h36A	4h36B	0	0	0	1	3o17	3alo	2	4	0	1
2yiy	2yiy	0	0	0	0	2dyl	3npc	3	3	0	1
3aln	3gc8	2	1	0	0	3brvB	1nfiE	0	0	2	2
1mod	1wgl	0	0	2	0	1oy3	1ikn	0	0	0	2
3mopC	3mopG	1	1	1	1	4hga	3kn6	0	2	1	1
1t2k	4fv7	0	0	0	0	1lb4	4g3d	3	0	2	0
3hcs	2j4o	1	4	3	1	2eva	2dyl	3	1	0	1
1nfiE	1nfiD	0	0	2	0	2hw6	3gc8	0	0	2	0
4depA	4depC	3	1	0	0	1wzy	1fos	2	2	1	0
4depB	4depC	1	0	0	3	2ac3	4fv0	0	1	0	1
2jrh	3fme	1	1	1	0	2mod	2z5v	2	1	0	0
3kn5	1tvo	2	2	1	0	3vut	3e7o	6	3	1	1
2y8oB	2y8oA	0	0	1	0	1ukh	1t2k	3	1	0	0
1s9i	4fv7	0	1	0	1	2y9qA	2y9qB	3	1	0	0
1svc	1oy3	0	0	1	0	3brtA	3brtB	0	0	3	2
1jnm	3npc	2	2	1	2	3vn9	3gp0	2	1	2	4
3mopK	3mopG	1	0	2	3	2zoq	1vzo	1	0	1	0
3hcs	3mopK	2	2	3	2	2pph	3vut	7	2	0	1
1dux	2baj	0	0	2	1	1w82	3aln	3	8	1	1
3elj	1dux	3	3	1	0	3fv8	2dyl	1	4	2	1
3tei	1nkp	1	5	0	0	3mopG	1lb5	0	5	1	1
1fosEG	1ukh	3	3	0	1	1nfiAC	3brtB	0	0	0	2
3gutB	1nfiB	0	0	0	0	2js7	1lb6	2	3	1	0
3gutB	3gutA	0	0	2	0	1t2k	3gp0	0	0	0	1
1t2k	3kn5	0	0	2	2	3aln	3fv8	2	3	1	0
2p55	4fv4	5	2	0	0	2eva	4dn5	3	5	0	1
1nfiAC	1oy3	0	0	3	1	2jbo	3gc9	2	0	1	2
3ody	2hw6	0	0	0	2	1fosE	1fosF	3	3	1	2
3mop	1wgl	3	1	0	3	3que	1t2k	0	0	2	1
1nfiB	1nfiA	0	0	2	0	2l5x	4depC	1	2	4	1
2jbo	3q9q	2	2	2	7	1mod	2z5v	3	1	0	0
3kn5	1kv1	3	1	3	1	3alo	2yiy	7	3	1	0
3gc9	1vzo	1	0	2	2	2eva	3vn9	3	4	0	1
1fosF	2r9s	0	1	0	0	2onlA	2onlC	3	1	2	3
2zdu	1fosE	1	1	1	0	4depA	4depB	3	5	5	6
2eva	2eci	1	3	0	0	2l5x	4depB	3	2	4	8
2c60	2dyl	4	2	1	1						

**Table 3:** t-test for COSMIC mutations mapped onto the interface region including the nearby residues.

	*COSMIC mutations on interface region+nearby residues/total interface size with nearby residues*	*COSMIC mutations on noninterface region/total noninterface size*
Mean	0.039	0.061
Variance	0.001	0.002
Observations	77	77
Pooled Variance	0.002	
Hypothesized Mean Difference	0	
df	152	
t Stat	−3.397	
P(T< = t) one-tail	0.0004	
t Critical one-tail	1.655	
P(T< = t) two-tail	**0.0009**	
t Critical two-tail	1.976	

**Table 4:** t-test for SNPs mapped onto the interface region including the nearby residues.

	*SNPs on interface region+nearby residues/total interface size with nearby residues*	*SNPs on noninterface region/total noninterface size*
Mean	0.026	0.047
Variance	0.001	0.002
Observations	77	77
Pooled Variance	0.001	
Hypothesized Mean Difference	0	
df	152	
t Stat	−3.932	
P(T< = t) one-tail	0.00006	
t Critical one-tail	1.655	
P(T< = t) two-tail	**0.00013**	
t Critical two-tail	1.976	

### Oncogenic mutations in the interfaces of MKK4 with JNK2 and JNK3

**Figure 4:**
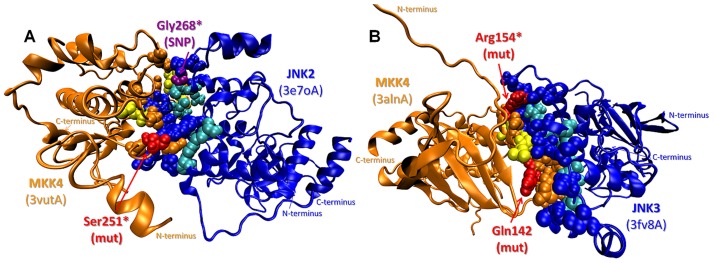
SNPs/mutations mapped on the predicted complexes of MKK4 with JNK2 and JNK3. **A.** MKK4 (mitogen-activated kinase kinase 4) - JNK2 (c-Jun N-terminal kinase 2) complex: Ser251 (p.Ser251Asn mutation - metastatic melanoma) on MKK4 and Gly268 (p.Gly268Ala SNP) on JNK2 are on the interface **B.** MKK4-JNK3 complex: Gln142 residue (p.Gln142Leu mutation - lung carcinoma) is a nearby residue of the interface and Arg154 residue (p.Arg154Trp mutation - colorectal adenocarcinoma) is on the interface as a computational hot spot residue. Orange and blue balls: interface residue atoms on MKK and JNK, respectively; yellow and cyan balls: hot spot residue atoms on MKK and JNK, respectively; red and purple balls: interface* and nearby residue atoms related to SNP and mutations on MKK and JNK, respectively.

Three oncogenic mutations and a SNP are observed either directly on the predicted interfaces of MKK4 with JNK2 and JNK3 (the SNP is a computational hot spot residue in the MKK4-JNK3 interaction) or as nearby residues (Figure 4). In Figure 4A, the predicted MKK4-JNK2 interaction has a binding energy of −12.29 energy units. Analysis of interface residues of the predicted complex reveals that MKK4 Ser251 and JNK2 Gly268 correspond to the interface region. These residues are important because the p.Ser251Asn mutation is involved in metastatic melanoma and p.Gly268Ala is a SNP. Figure 4B shows the predicted complex of MKK4-JNK3. On MKK4, Arg154 is mapped to the interface as a computational hot spot residue and its mutation to Tryptophan (p.Arg154Trp) is involved in colorectal adenocarcinoma. Moreover, Gln142 is a nearby residue of the interface and the MKK4 p.Gln142Leu mutation is involved in lung squamous cell carcinoma. Although it is known that MKK4 interacts with MAPKs (JNK1/2/3 and p38α/β) through its MAPK-docking site (D-site) [42], here we identify a complementary binding interface between MKK4 and JNK2/3. The D-site is located at the N-termini of MKK4 (residues 37–52), but the N-termini is missing in all of the 3D structures (PDB codes: 3aln, 3alo and 3vut; including residues between 80 and 399) and hence could not be included in our predictions. However, we modeled a complementary interface, which is not at the D-site but in the kinase catalytic domain. This model can be explained by the finding that although the D-site facilitates the activation of MAPKs through MKK-MAPK signaling, the specificity is also affected by allosteric cooperativity among other binding motifs in the kinase catalytic domains [43]. The mutations involve important residues in the critical domains of MKK4 such as Ser251 in the activation loop and Gln142 in the αC-helix. Gln142 was also proposed by another study [30] to be in the interface of the MKK4-human kinase STK4 interaction and the p.Gln142Leu mutation is speculated to switch-off native partners, gathering non-native interaction partners instead. Also, the MKK4 protein has a large interface for the dimer formed in the experimentally identified crystal structure (PDB code: 3alnAB) which includes Gln142. Similar to the dimer in the PDB, in our predictions both MKK4 and JNK3 interact with each other through their kinase domains, forming a large interface area including Gln142 as a nearby residue. Similarly, a putative interface of MKK-MAPK interaction was found to overlap with the ERK2 dimerization interface [44]. Wilsbacher *et al.* also listed the important regions on the MAPK for MKK-MAPK binding; one of them is the tip of the C helix [44], which is in accordance with our model as Gln142 in the αC-helix of MKK4 is one of the mutants and found to be important in MKK4-JNK3 interaction. In short, the predicted interaction does not show a simple recognition (D-site) binding but rather involves a specificity binding model.

### The mechanisms of the MKK4 mutations

**Figure 5:**
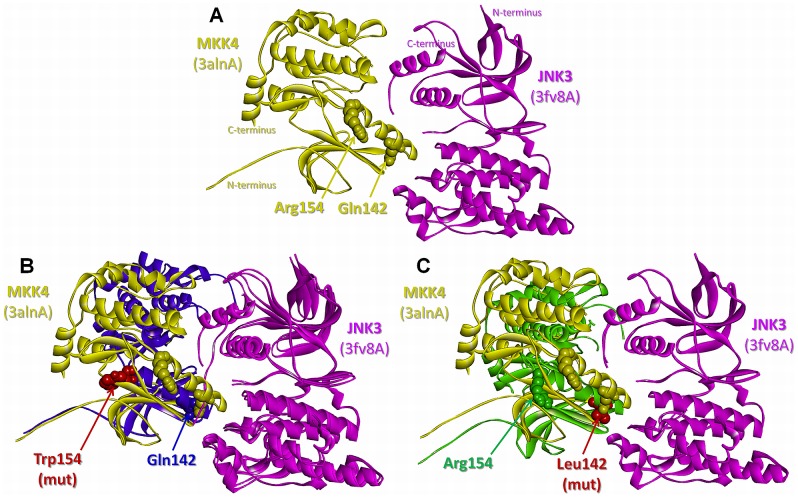
The effects of p.Arg154Trp and p.Gln142Leu mutations on the predicted structure of the MKK4-JNK3 complex. **A.** The predicted structure of the wild-type MKK4-JNK3 complex with predicted binding energy of −12.66 energy units. **B.** The predicted structure of the mutant MKK4-JNK3 complex (Arg154Trp) with predicted binding energy of −12.84 energy units aligned with the wild-type complex. **C.** The predicted structure of the mutant MKK4-JNK3 complex (Gln142Leu) with predicted binding energy of −41.14 energy units aligned with the wild-type complex. Yellow/purple/green and pink balls: residue atoms on MKK4 and JNK3, respectively; red balls: mutated residue atoms.

In order to better understand the mechanism through which the mutations may relate to cancer, these residues are mutated computationally and the resulting predicted binding energies of the complexes are compared (Table 5). After individual energy minimization of the target structures and re-running PRISM on the energetically minimized wild type and mutant targets, we observe models of protein-protein complexes with slightly different interface residues and conformations for the minimum energy solutions. The comparisons are based on these reference interactions. For the MKK4-JNK3 interaction, results are also obtained using a different template (1p4oAB) than the original (2ab0AB). None of the complexes predicted using the original template is favorable due to the predicted positive binding energy values after the minimization and we are unable to assess mutations based on unfavorable reference interaction. However, predictions of the MKK4-JNK3 interaction based on the 1p4oAB template clearly show the effect of p.Gln142Leu and p.Arg154Trp mutations as the wild type complex is still favorable after the minimization (−12.66 energy units, Table 5 and Figure 5A). The mutation of Arg154 to Tryptophan abolishes the interaction as the predicted binding energy becomes positive (12.84 energy units), implying that the interaction is not favorable anymore (Figure 5B). We can explain this change in terms of the specificity as an effect of a loss-of-function type mutation, as MKK4 cannot phosphorylate and activate JNK3 and its tumor-suppression function is lost. On the other hand, Gln142 is a nearby residue and its mutation to Leucine causes a dramatic decrease in the predicted binding energy (−41.14 energy units) with respect to the newly predicted reference MKK4-JNK3 interaction (−12.66 energy units) (Table 5 and Figure 5C). We observed that the interaction not only still takes place after the mutation occurs but also it is predicted to have a tighter binding.

**Table 5:** The effect of mutations on the MKK4-JNK3, MKK7-JNK3 and IL1A(IL-1α)-IL1R1 interactions.

Interacting Proteins		Template Interface	Energy
JNK3 (3fv8A)-wt	MKK4 (3alnA)-wt	1p4oAB	−12.66*
JNK3 (3fv8A)-wt	MKK4 (3alnA)-p.Arg154Trp	1p4oAB	12.84
JNK3 (3fv8A)-wt	MKK4 (3alnA)-p.Gln142Leu	1p4oAB	−41.14

Interacting Proteins		Template Interface	Energy
JNK3 (3fv8A)-wt	MKK7 (2dylA)-wt	1ftaAC	−11.66*
JNK3 (3fv8A)-wt	MKK7 (2dylA)-p.Arg178Cys	1ftaAC	31.03
JNK3 (3fv8A)-wt	MKK7 (2dylA)-p.Arg178His	1ftaAC	45.40

Interacting Proteins		Template Interface	Energy
IL1A (2l5xA)-wt	IL1R1(4depB)-wt	1itbAB	−19.5*
IL1A (2l5xA)-p.Asp26Asn (SNP)	IL1R1(4depB)-wt	1itbAB	−27.71
IL1A (2l5xA)-p.Asn29Ser (SNP)	IL1R1(4depB)-wt	1itbAB	−33.67
IL1A (2l5xA)-p.Arg34Gln (SNP)	IL1R1(4depB)-wt	1itbAB	−31.87
IL1A (2l5xA)-p.Asp64His (SNP)	IL1R1(4depB)-wt	1itbAB	−24.46
IL1A (2l5xA)-p.Ile68Asn (SNP)	IL1R1(4depB)-wt	1itbAB	-
IL1A (2l5xA)-p.Ile154Arg (SNP)	IL1R1(4depB)-wt	1itbAB	−35.65
IL1A (2l5xA)-p.Gly117Ser (Mut)	IL1R1(4depB)-wt	1itbAB	−30.11
IL1A (2l5xA)-wt	IL1R1(4depB)-p.Pro116Leu (SNP)	1itbAB	−38.02
IL1A (2l5xA)-wt	IL1R1(4depB)-p.Asn204Ser (SNP)	1itbAB	−42.52
IL1A (2l5xA)-wt	IL1R1(4depB)-p.Gln236Lys (SNP)	1itbAB	−24.03
IL1A (2l5xA)-wt	IL1R1(4depB)-p.Ser263Thr (SNP)	1itbAB	−30.88
IL1A (2l5xA)-wt	IL1R1(4depB)-p.Val264Glu (SNP)	1itbAB	−21.89
IL1A (2l5xA)-wt	IL1R1(4depB)-p.Ile303Val (SNP)	1itbAB	−26.67
IL1A (2l5xA)-wt	IL1R1(4depB)-p.Glu202Ala (Mut)	1itbAB	−21.10
IL1A (2l5xA)-wt	IL1R1(4depB)-p.Asp254Asn (Mut)	1itbAB	−43.31
IL1A (2l5xA)-wt	IL1R1(4depB)-p.Ile276Thr (Mut)	1itbAB	−34.72
IL1A (2l5xA)-wt	IL1R1(4depB)-p.Lys298Arg (Mut)	1itbAB	−23.87
IL1A (2l5xA)-p.Ile68Asn (SNP)	IL1R1(4depB)-p.Pro116Leu (SNP)	1itbAB	251.84
IL1A (2l5xA)-p.Ile68Asn (SNP)	IL1R1(4depB)-p.Asn204Ser (SNP)	1itbAB	405.47
IL1A (2l5xA)-p.Ile68Asn (SNP)	IL1R1(4depB)-p.Gln236Lys (SNP)	1itbAB	-
IL1A (2l5xA)-p.Ile68Asn (SNP)	IL1R1(4depB)-p.Ser263Thr (SNP)	1itbAB	-
IL1A (2l5xA)-p.Ile68Asn (SNP)	IL1R1(4depB)-p.Val264Glu (SNP)	1itbAB	−3.37
IL1A (2l5xA)-p.Ile68Asn (SNP)	IL1R1(4depB)-p.Ile303Val (SNP)	1itbAB	689.08
IL1A (2l5xA)-p.Ile68Asn (SNP)	IL1R1(4depB)-p.Glu202Ala (Mut)	1itbAB	688.03
IL1A (2l5xA)-p.Ile68Asn (SNP)	IL1R1(4depB)-p.Asp254Asn (Mut)	1itbAB	−7.9
IL1A (2l5xA)-p.Ile68Asn (SNP)	IL1R1(4depB)-p.Lys298Arg (Mut)	1itbAB	−6.01
IL1A (2l5xA)-p.Ile68Asn (SNP)	IL1R1(4depB)-p.Ile276Thr (Mut)	1itbAB	−40.64

The wild type target structures are energetically minimized, related residues are mutated and PRISM is re-run to predict the protein-protein interactions.

* The new reference interactions for comparisons are the predicted interactions between energetically minimized wild type targets and for the three cases they are: MKK4-JNK3 interaction predicted using 1p4oAB template with a predicted binding energy of −12.66 energy units; MKK7-JNK3 predicted using 1ftaAC template with a predicted binding energy of −11.66 energy units; and IL1A-IL1R1 predicted using 1itbAB template with a predicted binding energy of −19.5 energy units.

The contribution of this change to cancer development can be interpreted in two ways: a stronger complex between MKK4 and JNK3 takes place and the interaction, previously transient, gets so strong that it becomes obligate and cannot dissociate so that the activated JNK3 cannot activate its downstream targets, resulting in tumor in predisposed persons; or the essentially constitutively active MKK4 phosphorylates the JNK3 targets and the elevated, unregulated JNK activation leads to tumors as shown previously [45]. A similar case was also previously shown for EGFR, the activation loop of which could not switch to the inactive conformation due to a mutation. Hashimoto *et al.* showed that single cancer mutations in kinase domains destabilized inactive states [46]. Moreover, our structural analysis explains the experimental data in the study of Ahn *et al.* [18], who performed site-directed mutagenesis using human *MAP2K4* (*MKK4*) cDNA in order to create somatic mutants which are subjected to a mutant JNK1 as a substrate and concluded that p.Arg154Trp is one of the loss-of-function mutations whereas p.Gln142Leu is a gain-of-function mutation which results in a highly active MKK4 similar to a synthetic constitutively active mutant.

### Oncogenic mutations on the interactions of MKK7 with JNK3

In the second case study, the analysis of the predicted MKK7-JNK3 complex reveals the effect of the p.Arg178Cys and p.Arg178His mutations in MKK7. Similarly, the individual targets are energetically minimized, *in silico* mutations are performed, and PRISM is re-run to obtain new values of predicted binding energies (Table 5). When the values are compared to that of the new reference MKK7-JNK3 interaction (−11.66 energy units), a jump in both mutant cases (Table 5) and a significant change in the conformations are observed, making the energies positive so that these interactions cannot take place anymore. Thus, the mutation of Arg178 to Cys and His are both inferred as loss-of-function mutations abrogating the interaction with the JNK3 partner. Since MKK7 is a tumor suppressor, the inhibition mechanism of the MKK7-JNK3 interaction could presumably lead to tumor progression due to the loss-of-function of MKK7.

### The interactions of IL-1 receptors IL1R1 and IL1RAP

Finally, we concentrate on two IL-1 pathway specific interactions, namely the interactions of IL-1 with receptors IL1R1 and IL1RAP. After mapping the oncogenic mutations and SNPs onto the interfaces of these receptors, we observe that the predicted structure for IL-1α-IL1R1 interaction is important due to containing 11 SNPs and 5 mutations on its interface and nearby residues (see the last row of Table 2). Comparison of the predicted binding interface to experimentally reported interactions reveals common residues, confirming the predicted complex structure of IL-1α-IL1R1. In the work of Labriolatompkins *et al.* [47], using oligonucleotide-directed mutagenesis, they determined seven important residues on the binding site of human IL-1α for IL1R1 binding. When we mapped these residues on the PDB structures and compared them to the interface of the predicted wild type IL-1α-IL1R1 interaction (with −71.92 units of predicted binding energy), we observed that 4/7 residues (Arg16, Ile18, Ile68, Trp113) are in common and the remaining three residues (Asp64, Asp65, Lys100) are nearby residues of the interface.

**Figure 6:**
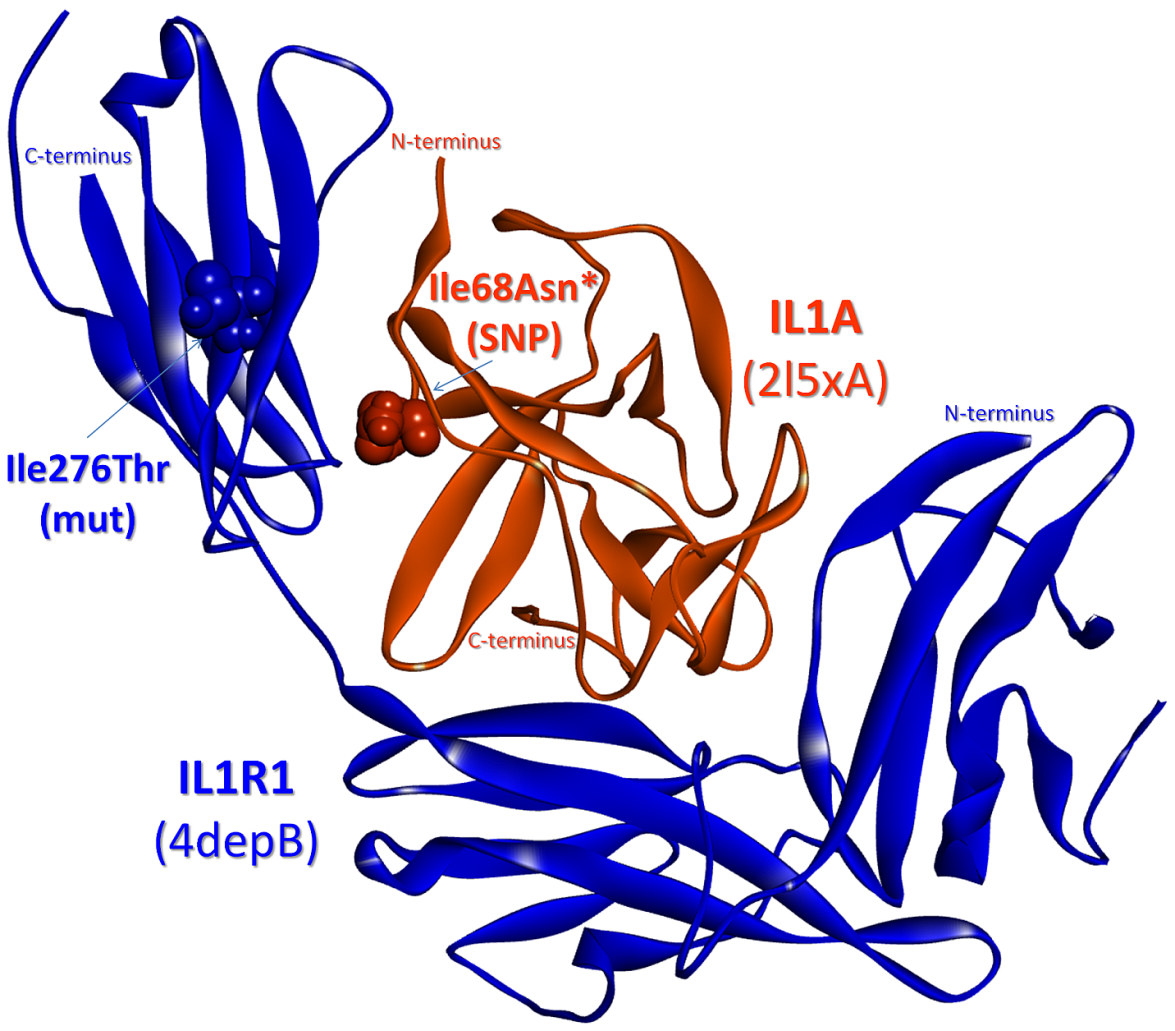
SNPs/mutations mapped on the predicted complex of IL-1α with IL1R1. IL1A (Interleukin 1 alpha) – IL1R1 (Interleukin 1 type I receptor) complex: Ile 68 (p.Ile68Asn SNP) is on the interface as a computational hot spot residue* and Ile276 (p.Ile276Thr mutation - endometrioid carcinoma) is a nearby residue for the interface. Orange and blue balls: residue atoms related to the SNP and the mutation on IL1A and IL1R1, respectively.

After confirming that the predicted structure is in accordance with related data in the literature, the binding energies of the mutants are compared to the energetically minimized reference interaction (−19.5 energy units). Being a ligand-receptor interaction, this complex has a large interface containing 75 residues in addition to 104 nearby residues. Intuitively, we expect to observe insignificant effects of SNPs or single point mutations on the interaction. The results are in parallel with our expectation and there are no significant changes in the predicted binding energies for the interactions with mutant proteins. However, we notice an exception for the p.Ile68Asn SNP on IL-1α ligand (Table 5). This SNP is observed to be very important for binding as it blocks the interaction of the mutant ligand not only with the wild type receptor but also with most of the mutant receptors (Table 5). This observation may be supported by the information that Ile68 is both a computational hot spot residue and experimentally determined as critical in binding [47]. Thus, importantly, this SNP may affect the innate immune system and inflammatory response as this interaction is critical for the initiation of IL-1 signaling, with the only alternative pathway being the IL1B(IL-1β)-IL1R1 interaction. However, interestingly this is not always the case. A ligand bearing the p.Ile68Asn substitution (a computational hot spot) appears to interact with a p.Ile276Thr mutant receptor (a nearby residue of the interface) with the energy of −40.64, suggesting that this mutation on the receptor is in fact useful for canceling out the effect of the SNP on the ligand (Figure 6). Although these two residues are not in contact in three dimensional space (with a distance of 16.1 Å between them), the change in the binding energy is significant implying that the difference stems from slight conformational changes. This observation reveals that compensatory changes do not necessarily involve residues in contact. The p.Ile276Thr mutation on IL1R1 is important: according to the COSMIC database [48], [49] it is related to endometrioid carcinoma and IL1R1 expression was found to be increased in active endometriotic lesions [50]. This fact supports our finding that IL-1α-IL1R1 interaction is still favorable between this mutant receptor and IL-1α with the p.Ile68Asn SNP, and emphasizes the usefulness of our strategy.

To conclude, in the absence of experimental data identifying the location of the mutations and SNPs on the protein structures, we are able to computationally clarify the mechanism of inhibition/activation by mapping these mutations on the interface of the predicted MKK4-JNK3, MKK7-JNK3 and IL-1α-IL1R1 complexes; and our account is corroborated by available experimental data.

### Conclusions

Here, we reconstructed the IL-1 signaling pathway by combining related pathways and information in the literature, expanding the previously constructed structural apoptosis pathway. By predicting protein-protein complexes throughout the pathway using the PRISM algorithm, the structural coverage of the pathway increased up to 71%. The distributions of oncogenic mutations and SNPs in the predicted structures of protein-protein complexes indicated that they significantly correspond to interface and adjoining residues, and more importantly, in some cases to computational hot spot residues. While oncogenic mutation and SNP data are reported for single proteins, by mapping them onto interfaces we are able to determine the critical binding partners, interactions with whom are affected by the mutations. Additionally, *in silico* mutagenesis of the corresponding residues and comparison of the change in the binding energies between the wild type and mutant shed light on the mechanism of cancer development, inflammation and other potential diseases. The IL-1α-IL1R1 interaction bearing the p.Ile68Asn and p.Ile276Thr mutations provides one remarkable example in inflammatory response and cancer.

## Materials and Methods

The reconstructed IL-1 signaling pathway (Figure 2) is used as the target pathway for predicting protein-protein interactions. There are 54 nodes (proteins) and 129 edges (interactions) between them (Tables S3 and S4). Four nodes (IRAK1, MEKK1, MKK3 and TAB3) are excluded as they do not have experimentally identified 3D structures. 104 edges link the remaining target proteins (50 nodes), and only 15 of these have structures of protein-protein complexes in the PDB (Figures 3A and S1, Tables S1, S3 and S4). Model structures are used for the TIR domains of IL1R1 and IL1RAP receptors as they are the key receptors initiating the signaling pathway and only the structures of their extracellular domains have been deposited in the PDB. The theoretical model structures are obtained from ModBase which is a database of comparative protein structure models [51].

### Protein-protein interaction prediction algorithm – PRISM

**Figure 7:**
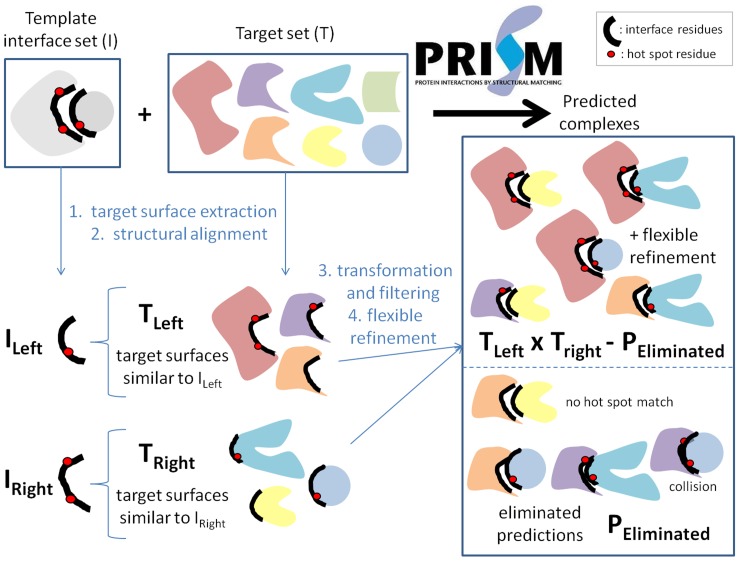
The PRISM algorithm flow. Two sets are given as the input: template and target. Four consecutive steps are executed to produce the output set which is composed of the structures of protein-protein complexes predicted to have the lowest binding energies. In this figure, the template set contains only one member for visualization simplicity, but it is important to note that the default template set of the algorithm is composed of 7922 interface members.

The 3D structures of protein-protein complexes in the reconstructed IL-1 signaling pathway are predicted by using the PRISM (PRotein Interactions by Structural Matching) [38]–[40] tool (Figure 7). PRISM is a large-scale protein-protein interaction prediction, modeling and structure assembly tool [40] that previously was successfully applied to several pathways [37], [52]–[54]. Two input sets, the template and the target sets, are required by the PRISM algorithm to obtain protein-protein interaction predictions (Figure 7). The structurally nonredundant (nonhomologous) unique interface dataset described by Tuncbag et al. [55] is used as the template set in this work. The dataset was generated by hierarchical clustering of 49512 two-chain interfaces (extracted from all of the available PDB complexes in the version of February 2006) into 8205 clusters. In this work, one representative interface for each of the clusters is combined into the template set. However, the number decreases from 8205 to 7922 due to the update in the PDB (since 2006) and replacement of previous structures with the better ones under different PDB codes. PRISM is a prediction algorithm based on three-dimensional protein structures and therefore it can only be applied to target proteins with known (experimental or high quality modeled) structures. The target set may contain a minimum of two proteins and the number of proteins in the set can increase up to any desired number, that is, all the proteins in a given pathway. Here, the target set is composed of IL-1 related PDB chains, the interactions and complex structures of which we want to predict. We focused on every single one of these and applied the prediction algorithm for each of the binary interactions (104 edges) between the targets that have 3D structures (50 nodes) (Table S3). However, for one interaction there might be more than two structures in the target set as each of the proteins may have more than one experimentally identified structure. We included all available PDB codes for the target proteins in each run to have a complete picture of possible protein-protein complexes.

The prediction algorithm has four main steps: target surface extraction, structural alignment of the template interface and the targets, transformation of the targets onto the template and eliminating collisions, and flexible refinement of the resulting complexes (Figure 7). Firstly, the surface residues of target chains are extracted via the Naccess program [56] which calculates the accessible surface area of residues. The criterion for a residue to be accepted as a surface residue is that its relative surface accessibility should be greater than 5%. In the second step, the structural comparison of the template interface chains with the target chain surfaces are carried out using the MultiProt structural alignment tool [57] based on some filters. For example, if the template chain has less than or equal to 50 residues, then 50% of the template residues should match the target surface residues; if larger than 50, a 30% match of template to target residues is required. In addition, at least one ‘hot spot’ residue on the template interface should match one of the hot spots on the target surface. In the third step, the resulting set of target surfaces from the previous set are transformed onto the corresponding template interfaces to form a complex so that these two targets are potential partners interacting with each other through an interface similar to the template interface architecture. Following transformation, PRISM eliminates the residues of the target chains that collide with each other. Finally, the FiberDock algorithm [58], [59] is used to refine the interactions allowing some flexibility, to resolve steric clashes of side chains, to compute the global energy of the complex and to rank the solutions based on the calculated binding energies. The calculated binding score is correlated with the experimental binding free energy and taken as the approximation of the binding free energy function [58], [60]. We accept a threshold of −10 energy units for a favorable interaction [54]. The detection of computational hot spot residues that are used in the analyses is done by using HotRegion Server [61].

### Computational analysis of the SNPs and mutations on the structures of the protein-protein complexes

Once all possible structures of protein-protein complexes in the IL-1 signaling pathway are predicted, the analysis of the interfaces in terms of SNP and mutation distribution is done. In addition to mapping the available SNPs and mutations onto predicted complexes and checking whether they correspond to the interface, *in silico* mutagenesis is also performed to observe the change in the predicted binding energy of the wild type and mutant complexes. The list of SNPs and mutations related to the target proteins is obtained from LS-SNP/PDB which is a web tool for genome-wide annotations of human non-synonymous SNPs mapped to Protein Data Bank structures [62] and COSMIC (Catalogue of Somatic Mutations in Cancer) database [48], [49], respectively. Based on the list, 394 SNPs and 535 oncogenic mutations are mapped onto the target PDB structures of the proteins in IL-1 pathway and these numbers decrease to 371 and 302, respectively when only the SNPs and mutations on the PDB surfaces are considered (Table 1). Then, these SNPs and mutations are mapped onto the interface residues and their nearby residues of both predicted and experimental interactions in the IL-1 pathway (Table 2). The statistical significance of these mappings are calculated by applying t-test to the following two sets: the ratio of mutations and SNPs mapped on interface and nearby residues to total interface size including nearby residues versus the ratio of mutations and SNPs on a noninterface surface region to total noninterface surface region (Tables 3 and 4). These ratios are calculated for each of the 77 interactions listed in Table 2. Note that the surface residues of target chains are extracted via Naccess program [56] as described in the previous subsection.

To understand the effect of oncogenic mutations and SNPs, those predicted to be at the interface (or nearby residues) are mutated in case studies using the FoldX plugin [63] for the YASARA molecular viewer [64]. There are two steps for computationally mutating the residues: energy minimization and residue change. The “RepairPDB” module of YASARA is used for energy minimization both before and after the residue change and the “BuildModel” module is used for mutation. For setting the FoldX options, default values are used except temperature, which is taken the same as the experimental temperature of the PDB structure. After obtaining the mutant structures, an energy minimization process is performed for the second time and the resulting structures are saved as PDB files and used as new targets for re-running the interaction prediction algorithm PRISM. The predicted binding energy values of the interactions are then compared to the reference values (Table 5).

## Supporting Information

Figure S1 Experimentally determined structures of protein-protein complexes mapped to the IL-1 signaling pathway. The PDB codes used in the figure are: IL1β-IL1R1-IL1RAP, 4dep; MYD88-IRAK2-IRAK4, 3mop; IKKα/IKKβ-IKKγ, 3brt; MKK6-MAPKp38α, 2y8o; MAPKp38α-MAPKAPK2, 2onl; JNK3-ATF2, 4h36; ERK2-Mnk1, 2y9q; c-Jun-c-Fos, 1fos; NF-κBp105-NF-κBp65, 3gut; NF-κBp50-NF-κBp65-IκBα, 1nfi. The blue color represents the proteins that precede its partners in the information flow.
(TIF)

Table S1 Verification of the predicted interactions.
(DOCX)

Table S2 The distribution of Mutations and SNPs on PDB Structures with PDB structures.
(DOCX)

Table S3 Experimental and computational information for the edges, linking proteins with 3D structures in PDB, in IL-1 network (104).
(DOCX)

Table S4 Experimental information for the edges (25) linking proteins, at least one of which does not have a 3D structure in PDB.
(DOCX)
