# Supplementary figures and images for: The Structural Pathway of Interleukin 1 (IL-1) Initiated Signaling Reveals Mechanisms of Oncogenic Mutations and SNPs in Inflammation and Cancer
Source: PLoS Comput Biol. 2014 Feb 13;10(2):e1003470. doi: 10.1371/journal.pcbi.1003470 (PMC3923659; doi:10.1371/journal.pcbi.1003470)

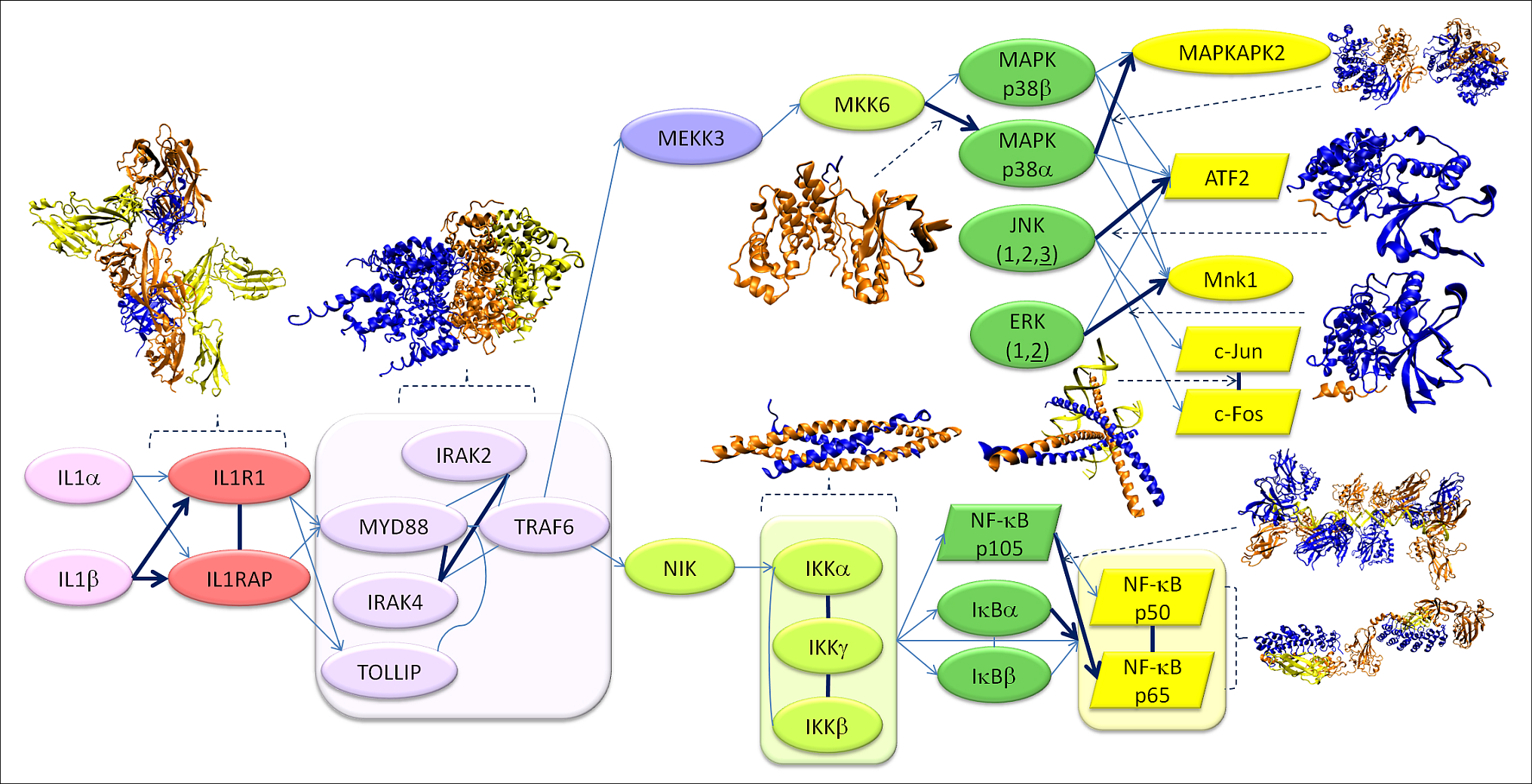

Supplement: Figure S1 — Experimentally determined structures of protein-protein complexes mapped to the IL-1 signaling pathway. The PDB codes used in the figure are: IL1β-IL1R1-IL1RAP, 4dep; MYD88-IRAK2-IRAK4, 3mop; IKKα/IKKβ-IKKγ, 3brt; MKK6-MAPKp38α, 2y8o; MAPKp38α-MAPKAPK2, 2onl; JNK3-ATF2, 4h36; ERK2-Mnk1, 2y9q; c-Jun-c-Fos, 1fos; NF-κBp105-NF-κBp65, 3gut; NF-κBp50-NF-κBp65-IκBα, 1nfi. The blue color represents the proteins that precede its partners in the information flow. (TIF) [file pcbi.1003470.s001.tif]
