# Supplementary material for: The Structural Pathway of Interleukin 1 (IL-1) Initiated Signaling Reveals Mechanisms of Oncogenic Mutations and SNPs in Inflammation and Cancer
Source: PLoS Comput Biol. 2014 Feb 13;10(2):e1003470. doi: 10.1371/journal.pcbi.1003470 (PMC3923659; doi:10.1371/journal.pcbi.1003470)
Supplement: Table S1 — Verification of the predicted interactions. (DOCX) [file pcbi.1003470.s002.docx]

**Table S1.** Verification of the predicted interactions with PDB structures

| **Interaction** | | **PDB Chains** | **Template Used in Prediction** | **Predicted Energy** | **Verification** |
| --- | --- | --- | --- | --- | --- |
| IL1b | IL1R1 | 4depAB | 1itbAB | -77.28 | Yes |
| IL1R1 | IL1RAP | 4depBC | 4depBC | -45.84 | Yes |
| IL1b | IL1RAP | 4depAC | 3o4oAB | -64.31 | Yes |
| NF-kBp105 | NF-kBp65 (NFKB3) | 3gutAB | 1my7AB | -111.25 | Yes |
| c-Jun | c-Fos | 1fosEF | 1sfkCD | -57.45 | Yes |
| IKKa/b | IKKg | 3brtAB | 2w4912 | -88.01 | Yes |
| NF-kBp50 | NF-kBp65 | 1nfiAB | 1my7AB | -124.37 | Yes |
| IkBa | NF-kBp65 | 1nfiAE | 1nfiCE | -78.15 | Yes |
| IkBa | NF-kBp50 | 1nfiDE | 3f8hAB | -15.86 | Yes |
| MAPKp38a | MAPKAPK2 | 2onlAC | 2onlAC | -78.01 | Yes |
| IRAK2 | IRAK4 | 3mopKG | 3mopJN | -25.62 | Yes |
| MYD88 | IRAK4 | 3mopFJ | 3mopFJ | -24.11 | Yes |
| JNK3 | ATF2 | 4h36AB |  |  | No interaction - Peptide |
| MKK6 | MAPKp38a (MAPK14) | 2y8oAB | - | - | No interaction - Peptide |
| ERK2 | MNK1 | 2y9qAB | - | - | No interaction - Peptide |
