# Supplementary material for: The Structural Pathway of Interleukin 1 (IL-1) Initiated Signaling Reveals Mechanisms of Oncogenic Mutations and SNPs in Inflammation and Cancer
Source: PLoS Comput Biol. 2014 Feb 13;10(2):e1003470. doi: 10.1371/journal.pcbi.1003470 (PMC3923659; doi:10.1371/journal.pcbi.1003470)
Supplement: Table S2 — The distribution of Mutations and SNPs on PDB Structures with PDB structures. (DOCX) [file pcbi.1003470.s003.docx]

**Table S2.** The distribution of Mutations and SNPs on PDB Structures

| Protein | PDB | COSMIC on PDB | COSMIC on PDB | COSMIC on PDB Surface | COSMIC on PDB Surface | LS-SNP on PDB | LS-SNP on PDB | LS-SNP on PDB Surface | LS-SNP on PDB Surface | Total PDB Residues | Total PDB Residues | Total Surface Residues | Total Surface Residues |
| --- | --- | --- | --- | --- | --- | --- | --- | --- | --- | --- | --- | --- | --- |
| ATF2, CREB2, ATF4 | 1t2kD | 1 | 1 | 0 | 0 | 1 | 1 | 1 | 1 | 61 | 61 | 61 | 61 |
| ATF2 | 4h36B | 0 | 0 | 0 | 0 | 0 | 0 | 0 | 0 | 8 | 8 | 8 | 8 |
| FOS, c-Fos | 1fosE | 4 | 4 | 4 | 4 | 2 | 2 | 2 | 2 | 60 | 60 | 60 | 60 |
| JUN, c-Jun | 1jnmA | 4 | 4 | 4 | 4 | 1 | 1 | 1 | 1 | 57 | 57 | 57 | 57 |
| JUN, c-Jun | 1fosF |  |  |  |  |  |  |  |  | 56 |  | 56 |  |
| MYC, c-Myc | 1nkpA | 8 | 8 | 7 | 7 | 0 | 0 | 0 | 0 | 88 | 88 | 81 | 81 |
| ELK1 | 1duxC | 7 | 7 | 5 | 5 | 0 | 0 | 0 | 0 | 88 | 88 | 61 | 61 |
| ERK1, MAPK3 | 2zoqA | 14 | 14 | 10 | 10 | 11 | 11 | 9 | 9 | 351 | 351 | 209 | 209 |
| ERK2, MAPK2 | 1wzyA | 26 | 26 | 15 | 17 | 3 | 3 | 3 | 3 | 350 | 365 | 207 | 199 |
| ERK2, MAPK2 | 3teiA | 25 |  | 15 |  | 3 |  | 3 |  | 339 |  | 203 |  |
| ERK2, MAPK2 | 4fv4A | 24 |  | 16 |  | 3 |  | 3 |  | 331 |  | 200 |  |
| ERK2, MAPK2 | 4fv7A | 26 |  | 17 |  | 3 |  | 3 |  | 343 |  | 202 |  |
| ERK2, MAPK2 | 1tvoA | 26 |  | 17 |  | 3 |  | 3 |  | 350 |  | 208 |  |
| ERK2, MAPK2 | 4fv0A | 26 |  | 17 |  | 3 |  | 3 |  | 343 |  | 202 |  |
| ERK2, MAPK2 | 2y9qA | 26 |  | 14 |  | 3 |  | 3 |  | 365 |  | 199 |  |
| histone H3.3 | 4hgaB | 0 | 0 | 0 | 0 | 6 | 6 | 6 | 6 | 93 | 93 | 85 | 85 |
| HSP27 | 3q9qA | 2 | 2 | 1 | 1 | 9 | 9 | 9 | 9 | 79 | 79 | 67 | 67 |
| NFKBIA | 1nfiE | 0 | 0 | 0 | 0 | 8 | 8 | 6 | 6 | 213 | 215 | 127 | 142 |
| NFKBIA | 1iknD |  |  |  |  |  |  |  |  | 215 |  | 142 |  |
| NFKBIB | 1oy3D | 0 | 0 | 0 | 0 | 0 | 0 | 0 | 0 | 220 | 220 | 130 | 130 |
| IKBKA, CHUK, IKBKB | 3brtA | 0 | 0 | 0 | 0 | 6 | 8 | 6 | 8 | 39 | 39 | 39 | 39 |
| IKBKB | 3brvA |  |  |  |  | 8 |  | 8 |  |  |  |  |  |
| IKBKG | 3brvB | 0 | 0 | 0 | 0 | 1 | 1 | 1 | 1 | 61 | 62 | 61 | 61 |
| IKBKG | 3brtB |  |  |  |  |  |  |  |  | 62 |  | 61 |  |
| IL1A | 2l5xA | 6 | 6 | 3 | 3 | 11 | 11 | 8 | 8 | 151 | 151 | 102 | 102 |
| IL1R1 | 4depB | 18 | 18 | 13 | 13 | 29 | 29 | 21 | 21 | 296 | 296 | 216 | 216 |
| IL1RAP | 4depC | 16 | 16 | 13 | 13 | 14 | 14 | 9 | 9 | 288 | 288 | 201 | 201 |
| IRAK2 | 3mopK | 3 | 3 | 1 | 1 | 11 | 11 | 5 | 5 | 93 | 93 | 65 | 65 |
| IRAK4 | 3mopG | 2 | 2 | 2 | 2 | 7 | 7 | 5 | 5 | 107 | 107 | 76 | 76 |
| IRF7 | 2o61A | 0 | 0 | 0 | 0 | 0 | 0 | 0 | 0 | 498 | 498 | 351 | 351 |
| JNK1, MAPK8 | 3o17A | 30 | 30 | 13 | 18 | 8 | 8 | 6 | 7 | 357 | 366 | 206 | 213 |
| JNK1, MAPK8 | 1ukhA | 26 |  | 14 |  | 8 |  | 7 |  | 321 |  | 195 |  |
| JNK1, MAPK8 | 3eljA | 30 |  | 18 |  | 8 |  | 6 |  | 366 |  | 213 |  |
| JNK2, MAPK9 | 3e7oA | 27 | 30 | 18 | 18 | 9 | 9 | 6 | 8 | 342 | 357 | 210 | 211 |
| JNK2, MAPK9 | 3npcA | 30 |  | 18 |  | 9 |  | 8 |  | 357 |  | 211 |  |
| JNK3 | 3fv8A | 1 | 2 | 0 | 1 | 8 | 8 | 4 | 4 | 333 | 356 | 201 | 203 |
| JNK3 | 2zduA | 1 |  | 0 |  | 8 |  | 4 |  | 330 |  | 198 |  |
| JNK3 | 2r9sA | 2 |  | 1 |  | 8 |  | 4 |  | 325 |  | 191 |  |
| JNK3 | 4h36A | 2 |  | 1 |  | 8 |  | 4 |  | 356 |  | 203 |  |
| MAPKAPK2 | 2jboA | 20 | 22 | 14 | 16 | 9 | 13 | 7 | 11 | 295 | 317 | 199 | 211 |
| MAPKAPK2 | 2onlC | 22 |  | 16 |  | 13 |  | 11 |  | 317 |  | 211 |  |
| MAPK14 | 1kv1A | 0 | 0 | 0 | 0 | 30 | 30 | 24 | 24 | 331 | 351 | 209 | 211 |
| MAPK14 | 2bajA | 0 |  | 0 |  | 30 |  | 24 |  | 337 |  | 203 |  |
| MAPK14 | 3odyX | 0 |  | 0 |  | 27 |  | 21 |  | 321 |  | 201 |  |
| MAPK14 | 3queA | 0 |  | 0 |  | 30 |  | 23 |  | 343 |  | 209 |  |
| MAPK14 | 1w82A | 0 |  | 0 |  | 30 |  | 24 |  | 351 |  | 211 |  |
| MAPK14 | 2y8oA | 0 |  | 0 |  | 30 |  | 24 |  | 343 |  | 208 |  |
| MAPK14 | 2onlA | 0 |  | 0 |  | 30 |  | 24 |  | 338 |  | 207 |  |
| MAPK11 | 3gc9A | 0 | 0 | 0 | 0 | 22 | 26 | 17 | 21 | 335 | 347 | 200 | 213 |
| MAPK11 | 3gp0A | 0 |  | 0 |  | 24 |  | 19 |  | 336 |  | 201 |  |
| MAPK11 | 3gc8A | 0 |  | 0 |  | 26 |  | 21 |  | 347 |  | 213 |  |
| MEKK3, MAP3K3 | 2pphA | 6 | 6 | 4 | 4 | 3 | 3 | 3 | 3 | 93 | 93 | 74 | 76 |
| MEKK3, MAP3K3 | 2jrhA | 6 |  | 4 |  | 3 |  | 2 |  | 93 |  | 76 |  |
| MEKK3, MAP3K3 | 2c60A | 5 |  | 4 |  | 1 |  | 1 |  | 83 |  | 64 |  |
| MKK1, MAP2K1 | 2p55A | 31 | 31 | 17 | 17 | 6 | 6 | 6 | 6 | 289 | 289 | 181 | 181 |
| MKK2, MAP2K2 | 1s9iA | 17 | 17 | 12 | 12 | 12 | 12 | 10 | 10 | 303 | 303 | 182 | 182 |
| MKK4, MAP2K4 | 3aloA | 56 | 56 | 40 | 40 | 6 | 6 | 4 | 4 | 289 | 289 | 190 | 190 |
| MKK4, MAP2K4 | 3alnA | 49 |  | 37 |  | 4 |  | 1 |  | 270 |  | 175 |  |
| MKK4, MAP2K4 | 3vutA | 37 |  | 25 |  | 3 |  | 2 |  | 203 |  | 138 |  |
| MKK6, MAP2K6 | 3vn9A | 25 | 25 | 18 | 19 | 7 | 7 | 6 | 6 | 291 | 291 | 174 | 174 |
| MKK6, MAP2K6 | 3fmeA | 25 |  | 19 |  | 6 |  | 5 |  | 268 |  | 184 |  |
| MKK6, MAP2K6 | 2y8oB | 0 | 0 | 0 | 0 | 0 | 0 | 0 | 0 | 8 | 8 | 8 | 8 |
| MKK7, MAP2K7 | 2dylA | 29 | 29 | 22 | 22 | 6 | 6 | 3 | 3 | 272 | 272 | 172 | 172 |
| MNK1, MKNK1 | 2hw6A | 0 | 0 | 0 | 0 | 16 | 16 | 10 | 10 | 242 | 242 | 145 | 145 |
| MNK1, MKNK1 | 2y9qB | 0 | 0 | 0 | 0 | 0 | 0 | 0 | 0 | 17 | 17 | 17 | 17 |
| MNK2, MKNK2 | 2ac3A | 0 | 0 | 0 | 0 | 9 | 9 | 7 | 7 | 277 | 277 | 191 | 191 |
| MSK1, RPS6KA5 | 3kn5A | 23 | 23 | 12 | 12 | 14 | 14 | 10 | 10 | 282 | 282 | 174 | 174 |
| MSK1, RPS6KA5 | 3kn6A | 23 |  | 12 |  | 14 |  | 10 |  | 282 |  | 172 |  |
| MSK1, RPS6KA5 | 1vzoA | 20 | 20 | 14 | 14 | 15 | 15 | 12 | 12 | 324 | 324 | 188 | 188 |
| MYD88 | 2js7A | 22 | 22 | 14 | 14 | 0 | 0 | 0 | 0 | 160 | 160 | 106 | 106 |
| MYD88 | 2z5vA | 20 |  | 14 |  | 0 |  | 0 |  | 141 |  | 89 |  |
| MYD88 | 3mopA | 7 | 7 | 7 | 7 | 0 | 0 | 0 | 0 | 105 | 105 | 74 | 74 |
| NF-kBp105 | 3gutB | 0 | 0 | 0 | 0 | 9 | 9 | 7 | 7 | 312 | 312 | 198 | 198 |
| NFKB1 | 1svcP | 0 | 0 | 0 | 0 | 8 | 8 | 6 | 6 | 311 | 311 | 198 | 198 |
| NFKB1 | 1nfiB | 0 |  | 0 |  | 2 |  | 2 |  | 107 |  | 76 |  |
| NFKB3, RELA | 1nfiA | 0 | 0 | 0 | 0 | 12 | 12 | 11 | 11 | 295 | 295 | 208 | 208 |
| NFKB3 | 3gutA |  |  |  |  | 8 |  | 7 |  | 273 |  | 184 |  |
| NIK, MAP3K14 | 4g3dA | 13 | 14 | 6 | 7 | 4 | 5 | 4 | 4 | 324 | 331 | 193 | 194 |
| NIK, MAP3K14 | 4dn5A | 14 |  | 7 |  | 5 |  | 4 |  | 331 |  | 194 |  |
| TAB1 | 2j4oA | 25 | 25 | 18 | 18 | 21 | 21 | 16 | 16 | 355 | 355 | 207 | 207 |
| TAB1&TAK1 | 2yiyA | 20 | 21 | 13 | 15 | 6 | 8 | 3 | 4 | 293 | 295 | 178 | 181 |
| TAK1 | 2evaA | 21 |  | 15 |  | 8 |  | 4 |  | 295 |  | 181 |  |
| TOLLIP | 1wglA | 3 | 3 | 3 | 3 | 5 | 5 | 3 | 3 | 59 | 59 | 51 | 51 |
| TRAF6 | 1lb4A | 11 | 12 | 9 | 11 | 3 | 3 | 2 | 2 | 155 | 155 | 112 | 112 |
| TRAF6 | 1lb6A | 12 |  | 9 |  | 3 |  | 2 |  | 155 |  | 108 |  |
| TRAF6 | 1lb5A | 12 |  | 11 |  | 3 |  | 2 |  | 155 |  | 106 |  |
| TRAF6 | 3hcsA | 13 | 13 | 11 | 11 | 9 | 9 | 7 | 7 | 157 | 157 | 119 | 119 |
| TRAF6 | 3hctA | 10 |  | 8 |  | 3 |  | 2 |  | 104 |  | 80 |  |
| TRAF6 | 2eciA | 7 |  | 5 |  | 2 |  | 1 |  | 86 |  | 74 |  |
| IL1B | 4depA | 16 | 16 | 12 | 12 | 4 | 4 | 2 | 2 | 151 | 151 | 100 | 100 |
| IL1R1 | 1mod | 0 | 0 | 0 | 0 | 0 | 0 | 0 | 0 | 157 | 157 | 106 | 106 |
| IL1RAP | 2mod | 0 | 0 | 0 | 0 | 0 | 0 | 0 | 0 | 145 | 145 | 96 | 96 |
|  | **TOTAL** |  | **535** |  | **354** |  | **394** |  | **299** |  | **10988** |  | **7181** |
