# Supplementary material for: The Structural Pathway of Interleukin 1 (IL-1) Initiated Signaling Reveals Mechanisms of Oncogenic Mutations and SNPs in Inflammation and Cancer
Source: PLoS Comput Biol. 2014 Feb 13;10(2):e1003470. doi: 10.1371/journal.pcbi.1003470 (PMC3923659; doi:10.1371/journal.pcbi.1003470)
Supplement: Table S3 — Experimental and computational information for the edges, linking proteins with 3D structures in PDB, in IL-1 network (104). (DOCX) [file pcbi.1003470.s004.docx]

**Table S3.** Experimental and computational information for the edges, linking proteins with 3D structures in PDB, in IL-1 network (104)

| **Interaction** | | **Experimental Evidence** | **PDB Structure** | **PRISM Prediction** |
| --- | --- | --- | --- | --- |
| IL1a | IL1R1 | BioGrid, InnateDB | - | + |
| IL1a | IL1RAP | - | - | + |
| IL1b | IL1R1 | BIND, BioGrid | + | + |
| IL1b | IL1RAP | InnateDB | + | + |
| IL1R1 | IL1RAP | BioGrid, InnateDB | + | + |
| IL1R1 | MYD88 | BioGrid, InnateDB, IntAct | - | + |
| IL1R1 | TOLLIP | BioGrid, InnateDB, IntAct | - | + |
| IL1RAP | MYD88 | BioGrid, InnateDB | - | + |
| IL1RAP | TOLLIP | BioGrid, InnateDB, IntAct | - | - |
| MYD88 | IRAK2 | BioGrid, InnateDB, IntAct, MINT | - | + |
| MYD88 | TRAF6 | BioGrid, InnateDB | - | + |
| MYD88 | IRAK4 | BioGrid, InnateDB, IntAct | + | + |
| IRAK2 | IRAK4 | BioGrid, InnateDB, IntAct | + | + |
| IRAK2 | TRAF6 | BioGrid, InnateDB, IntAct, MINT | - | - |
| IRAK4 | TRAF6 | BioGrid, InnateDB | - | - |
| MYD88 | TOLLIP | - | - | + |
| MYD88 | IRF7 | BIND, BioGrid, InnateDB | - | - |
| TRAF6 | IRF7 | BIND, BioGrid, InnateDB | - | + |
| TRAF6 | MEKK3 | BioGrid, IntAct, MINT | - | + |
| TRAF6 | NIK | BIND, BioGrid, IntAct, MINT | - | + |
| TRAF6 | TAK1 | BioGrid, IntAct, MINT | - | + |
| TRAF6 | TAB1 | BioGrid, IntAct, MINT | - | + |
| TRAF6 | TAB2 | BioGrid, InnateDB, IntAct | - | - |
| TRAF6 | TAB3 | BIND, BioGrid, InnateDB, IntAct | - | - |
| TAB1 | TAK1 | BIND, BioGrid, IntAct, MINT | - | + |
| TAK1 | TAB2 | BioGrid, IntAct, MINT | - | - |
| TAK1 | MKK6 | BioGrid, IntAct, MINT | - | + |
| TAK1 | MKK4 | BioGrid, IntAct, MINT | - | + |
| TAK1 | MKK7 | BioGrid, IntAct, MINT | - | + |
| MEKK3 | MKK6 | - | - | + |
| MEKK3 | MKK4 | BioGrid | - | + |
| MEKK3 | MKK7 | - | - | + |
| TAK1 | NIK | BioGrid, IntAct, MINT | - | + |
| TAK1 | IKKb | BioGrid, MINT | - | - |
| NIK | IKKa | BioGrid, IntAct, MINT | - | - |
| IKKa | IKKb | BioGrid, IntAct, MINT | - | + |
| IKKa | IKKg | BioGrid, IntAct, MINT | - | + |
| IKKg | IKKb | BioGrid | + | + |
| MKK6 | MAPKp38b | - | - | + |
| MKK6 | MAPKp38a | BioGrid, MINT | + | + |
| MKK4 | MAPKp38b | - | - | + |
| MKK4 | MAPKp38a | BioGrid, MINT | - | + |
| MKK4 | JNK1 | BIND, BioGrid, IntAct, MINT | - | + |
| MKK4 | JNK2 | BioGrid, IntAct, MINT | - | + |
| MKK4 | JNK3 | BioGrid, IntAct, MINT | - | + |
| MKK7 | JNK1 | BioGrid, IntAct | - | - |
| MKK7 | JNK2 | BioGrid | - | + |
| MKK7 | JNK3 | BioGrid | - | + |
| MKK1 | ERK1 | BioGrid | - | - |
| MKK1 | ERK2 | BioGrid | - | + |
| MKK2 | ERK1 | BioGrid | - | - |
| MKK2 | ERK2 | BioGrid | - | + |
| IKKa | NF-kBp105 | BioGrid | - | - |
| IKKb | NF-kBp105 | BioGrid | - | - |
| IKKg | NF-kBp105 | BioGrid | - | - |
| IKKa | IkBa | BioGrid, IntAct | - | - |
| IKKb | IkBa | BioGrid, IntAct, MINT | - | - |
| IKKg | IkBa | BioGrid, IntAct, MINT | - | + |
| IKKa | IkBb | BioGrid, IntAct | - | - |
| IKKb | IkBb | BioGrid, IntAct | - | - |
| IKKg | IkBb | BioGrid, IntAct | - | - |
| IKKa | NF-kBp65 | BioGrid, IntAct, MINT | - | - |
| IKKb | NF-kBp65 | BioGrid, MINT | - | - |
| IKKg | NF-kBp65 | - | - | + |
| IkBa | IkBb | BioGrid, IntAct | - | + |
| NF-kBp105 | NF-kBp50 | - | - | + |
| NF-kBp105 | NF-kBp65 | BioGrid, IntAct, MINT | + | + |
| IkBa | NF-kBp50 | BioGrid, IntAct | + | + |
| IkBa | NF-kBp65 | BioGrid, IntAct, MINT | + | + |
| IkBb | NF-kBp50 | IntAct | - | + |
| IkBb | NF-kBp65 | BioGrid, IntAct, MINT | - | + |
| NF-kBp50 | NF-kBp65 | BioGrid, IntAct, MINT | + | + |
| MAPKAPK2 | HSP27 | BioGrid | - | + |
| MSK1 | CREB | BioGrid | - | + |
| MSK1 | histoneH3 | - | - | + |
| Mnk1 | eIF-4e | BIND, MINT | - | - |
| c-Jun | c-Fos | BIND, BioGrid | + | + |
| MAPKp38a | MAPKAPK2 | BioGrid, InnateDB, IntAct, MINT | + | + |
| MAPKp38a | MSK1 | BioGrid, IntAct | - | + |
| MAPKp38a | ATF2 | BioGrid, InnateDB, MINT | - | + |
| MAPKp38a | Elk1 | - | - | + |
| MAPKp38a | MBP | - | - | - |
| MAPKp38a | Mnk1 | BioGrid, IntAct, MINT | - | + |
| MAPKp38b | MAPKAPK2 | IntAct | - | + |
| MAPKp38b | MSK1 | BioGrid | - | + |
| MAPKp38b | ATF2 | BIND, InnateDB, IntAct, MINT | - | + |
| MAPKp38b | Elk1 | - | - | - |
| MAPKp38b | MBP | - | - | - |
| MAPKp38b | Mnk1 | IntAct, MINT | - | + |
| JNK1 | ATF2 | BIND, BioGrid, IntAct, MINT | - | + |
| JNK1 | Elk1 | BIND | - | + |
| JNK1 | c-Jun | BIND, BioGrid | - | + |
| JNK1 | c-Fos | - | - | + |
| JNK2 | ATF2 | BIND, BioGrid, IntAct | - | - |
| JNK2 | Elk1 | - | - | - |
| JNK2 | c-Jun | BIND, BioGrid, IntAct, MINT | - | + |
| JNK2 | c-Fos | - | - | - |
| JNK3 | ATF2 | BioGrid | + | + |
| JNK3 | Elk1 | - | - | - |
| JNK3 | c-Jun | BioGrid, IntAct, MINT | - | + |
| JNK3 | c-Fos | - | - | + |
| ERK1 | MSK1 | - | - | + |
| ERK1 | ATF2 | BIND, BioGrid | - | - |
| ERK1 | Elk1 | BIND, BioGrid, IntAct | - | - |
| ERK1 | MBP | - | - | - |
| ERK1 | Mnk1 | BIND, BioGrid, MINT | - | - |
| ERK1 | c-Fos | BioGrid | - | - |
| ERK1 | c-Myc | BIND, BioGrid | - | - |
| ERK1 | Mnk2 | MINT | - | - |
| ERK2 | MSK1 | BioGrid | - | + |
| ERK2 | ATF2 | BioGrid, MINT | - | + |
| ERK2 | Elk1 | BioGrid, IntAct | - | - |
| ERK2 | MBP | BIND | - | - |
| ERK2 | Mnk1 | BIND, BioGrid, MINT | + | + |
| ERK2 | c-Fos | BIND, BioGrid | - | + |
| ERK2 | c-Myc | BioGrid | - | + |
| ERK2 | Mnk2 | BIND, BioGrid, IntAct, MINT | - | + |

* This table lists experimental evidence information for the edges; whether or not there is at least one PDB structure of that complex; and if PRISM predicted that edge or not.
