# Supplementary material for: The Structural Pathway of Interleukin 1 (IL-1) Initiated Signaling Reveals Mechanisms of Oncogenic Mutations and SNPs in Inflammation and Cancer
Source: PLoS Comput Biol. 2014 Feb 13;10(2):e1003470. doi: 10.1371/journal.pcbi.1003470 (PMC3923659; doi:10.1371/journal.pcbi.1003470)
Supplement: Table S4 — Experimental information for the edges (25) linking proteins, at least one of which does not have a 3D structure in PDB. (DOCX) [file pcbi.1003470.s005.docx]

**Table S4.** Experimental information for the edges (25) linking proteins, at least one of which does not have a 3D structure in PDB

| **Interaction** | | **Experimental Evidence** |
| --- | --- | --- |
| IRAK1 | IL1R1 | BioGrid, InnateDB, IntAct |
| IRAK1 | IL1RAP | BioGrid, InnateDB |
| IRAK1 | MYD88 | BioGrid, InnateDB, IntAct, MINT |
| IRAK1 | IRAK2 | BioGrid, InnateDB, IntAct, MINT |
| IRAK1 | IRAK4 | BioGrid, InnateDB, IntAct |
| IRAK1 | TOLLIP | BioGrid, InnateDB, IntAct |
| IRAK1 | TRAF6 | BIND, BioGrid, InnateDB, IntAct, MINT |
| MEKK1 | TRAF6 | - |
| MEKK1 | MKK3 | BAR |
| MEKK1 | MKK6 | - |
| MEKK1 | MKK4 | BioGrid,IntAct, MINT |
| MEKK1 | MKK7 | BioGrid,MINT |
| MEKK1 | MKK1 | BioGrid |
| MEKK1 | IKKa | BioGrid |
| MEKK1 | IKKb | BioGrid, IntAct |
| MEKK1 | IKKg | BioGrid |
| MKK3 | MEKK3 | BioGrid |
| MKK3 | TAK1 | BIND |
| MKK3 | MAPKp38a | BioGrid,IntAct, MINT |
| MKK3 | MAPKp38b | - |
| TPL2 | MKK1 | BioGrid |
| TPL2 | MAPKp38a | - |
| TPL2 | MAPKp38b | - |
| TPL2 | ERK1 | - |
| TPL2 | ERK2 | - |
| TPL2 | NF-kBp105 | BioGrid |
| TPL2 | IKKb | - |
